# Cognitive, functional and behavioral assessment: Alzheimer’s disease

**DOI:** 10.1590/S1980-57642011DN05030003

**Published:** 2011

**Authors:** Márcia L.F. Chaves, Claudia C. Godinho, Claudia S. Porto, Leticia Mansur, Maria Teresa Carthery-Goulart, Mônica S. Yassuda, Rogério Beato, Group Recommendations in Alzheimer’s Disease and Vascular Dementia of the Brazilian Academy of Neurology

**Affiliations:** 1 Neurology Service of the Hospital de Clínicas of Porto Alegre, Federal University of Rio Grande do Sul, Porto Alegre RS, Brazil; 2 Cognitive and Behavioral Neurology Group of the Department of Neurology of the University of São Paulo School of Medicine, Hospital das Clínicas of the University of São Paulo School of Medicine, and Center for Mathematics, Computing and Cognition, Federal University of ABC, São Paulo SP, Brazil; 3 Department of Physiotherapy, Speech Therapy and Occupational Therapy of the University of São Paulo School of Medicine, São Paulo SP, Brazil; 4 Department of Gerontology, School of Arts, Sciences and Humanities of the University of São Paulo (EACH-USP East), São Paulo SP, Brazil; 5 Research Group in Cognitive and Behavioral Neurology, Department of Internal Medicine, School of Medicine, Federal University of Minas Gerais (UFMG), Belo Horizonte MG, Brazil

**Keywords:** consensus, guidelines, functional assessment, cognitive evaluation, behavioral assessment

## Abstract

A review of the evidence on cognitive, functional and behavioral assessment for the diagnosis of dementia due to Alzheimer’s disease (AD) is presented with revision and broadening of the recommendations on the use of tests and batteries in Brazil for the diagnosis of dementia due to AD. A systematic review of the literature (MEDLINE, LILACS and SCIELO database) was carried out by a panel of experts. Studies on the validation and/or adaptation of tests, scales and batteries for the Brazilian population were analyzed and classified according to level of evidence. There were sufficient data to recommend the IQCODE, DAFS-R, DAD, ADL-Q and Bayer scale for the evaluation of instrumental activities of daily living, and the Katz scale for the assessment of basic activities of daily living. For the evaluation of neuropsychiatric symptoms, the Neuropsychiatric Inventory (NPI) and the CAMDEX were found to be useful, as was the Cornell scale for depression in dementia. The Mini-Mental State Examination has clinical utility as a screening test, as do the multifunctional batteries (CAMCOG-R, ADAS-COG, CERAD and MDRS) for brief evaluations of several cognitive domains. There was sufficient evidence to recommend the CDR scale for clinical and severity assessment of dementia. Tests for Brazilian Portuguese are recommended by cognitive domain based on available data.

## Abstract (pt)

Este artigo apresenta revisão e ampliação das recomendações sobre os testes e baterias empregados no Brasil para o diagnóstico e avaliação cognitiva, funcional e comportamental da demência na doença de Alzheimer (DA). De modo sistemático foi revista a literatura disponível (nas bases MEDLINE, LILACS e SCIELO) e os artigos foram avaliados e classificados por níveis de evidência, para se estabelecerem as recomendações. Para a avaliação funcional a recomendação é o uso das escalas IQCODE, DAFS-R, DAD, ADL-Q e Bayer para avaliação das atividades instrumentais da vida diária e escala Katz para avaliação das atividades básicas. Para avaliação dos sintomas neuropsiquiátricos foram recomendadas as escalas NPI e CAMDEX e a Cornell para depressão em demência. Como instrumento de rastreio deve-se utilizar o Mini-Exame do Estado Mental; quanto às baterias multifuncionais, pode-se aplicar CAMCOG-R, ADAS-COG, CERAD e MDRS, que avaliam brevemente várias funções cognitivas. Para avaliação clínica da demência e classificação de acordo com a gravidade é recomendada a escala CDR. São recomendados os testes por domínio cognitivo baseados nas evidências disponíveis para uso na nossa língua.

## Introduction

Dementia is a prevalent condition affecting an estimated 2.4 to 4.5 million individuals in the USA, depending on the criteria adopted.^1-3^ Furthermore, many older adults experience memory and other cognitive function impairment. Alzheimer’s disease (AD) is the most common cause of dementia, accounting for around 60% of cases of progressive cognitive impairment in the elderly population.^4^ Screening for AD before it is clinically detectable or during early stages of the disease is an extremely rational approach where interventions to prevent or delay the effects of the disease are available. However, the benefits of screening all asymptomatic elderly has neither been confirmed or refuted.^5^ Nonetheless, individuals reporting cognitive or cognition-related complaints should undergo comprehensive assessment. Physicians dealing with adults will encounter patients with memory impairments and should be prepared to properly assess them for causes of dementia.

Neuropsychological tests can screen for cognitive, behavioral and functional changes and assist physicians in the diagnostic process and planning of disease treatment and management strategies. As with all tests, neuropsychological assessment has its limitations and results must be interpreted set against other clinical, imaging and laboratory information. Neuropsychological assessments have the advantage of being objective, safe, portable and pertinent for measuring functional integrity of the brain. The results of neuropsychological assessments must be considered in the context of patient age, education, socioeconomic status and cultural background since these are known factors influencing performance on test batteries. In addition, technical and structural issues regarding the tests such as reliability, validity and sensitivity of assessment procedures have an impact on the conclusions reached in neuropsychological assessments.^6^

Neuropsychological testing is required in the diagnosis of Alzheimer’s disease by a number of prevailing diagnostic criteria (NINCDS-ADRDA, DSM-IV, CID-10) and is currently one of the main means of assessing the efficacy of drugs developed for the treatment of AD.

Physicians must perform assessments of patient mental status prior to referring them for neuropsychological tests to be carried out by trained professionals, where many clinics provide training on applying basic questionnaires.^7-9^ Screening tests however, produce a considerable rate of false-negative results, often failing to detect subtle cognitive changes and in many ways cannot substitute full neuropsychological testing.^10^

In 2006, the Scientific Department of Cognitive Neurology and Aging published recommendations for diagnosis, treatment, as well as cognitive and functional assessment.^11^ These recommendations are currently being updated by a panel of Brazilian experts in the field. A review of the evidence was performed by searching for relevant articles on the PUBMED, SCIELO and LILACS medical databases using key words elected for each module in order to retrieve data on national and international research. For the instruments assessed in this study, the availability of validation studies for the Brazilian population was considered an essential prerequisite given the influence of cultural and demographic aspects on performance in tests and scales. Therefore, only publications referring to Brazilian data were selected for analysis.

## Instrument selection

Instruments used in cognitive, behavioral and functional assessments were selected on the databases outlined, which adopted the appropriate criteria for validation studies of tests and scales, provided they were not characterized as clinical trials. The group was therefore based on the classification given in Tables 1 and 2.^12^

**Table 1:** Classification of evidence.^12^

Class	Description
I	Evidence derived from well-planned prospective study conducted in broad spectrum of individuals with the suspected condition, using "gold standard" for defining cases, where test has been applied in blinded manner and enables assessment of appropriate diagnostically accurate tests.
II	Evidence derived from well-planned prospective study conducted in limited spectrum of individuals with the suspected condition, or by well-planned retrospective study in broad spectrum of individuals with confirmed condition (using gold standard), compared with broad spectrum of control subjects, where tests have been applied in blinded manner, and enables measurement of appropriate diagnostically accurate tests.
III	Evidence derived from retrospective study in limited spectrum of individuals with the confirmed condition and control subjects, in which tests have been applied in blinded manner.
IV	Any design methodology in which test has not been applied in blinded mode or is drawn from evidence based exclusively on opinion of a specialist or on a descriptive casuistic (without controls).

**Table 2:** Definitions for evidence-based practice recommendations.^12^

Recommendation	Description
Standard	Principle for care of patient reflecting a high degree of clinical certainty (usually requires Class I evidence directly focused on the clinical issue, or indisputable evidence when circumstances preclude randomized clinical trials).
Norm	Recommendation for care of patient which reflect a moderate degree of clinical certainty (usually requires Class II evidence or strong consensus on Class III evidence).
Practice option	Strategy for patient care of clinically uncertain use (inconclusive or evidence or conflicting opinions).
Suggestion	Practice recommendation for recently approved or emerging technologies or therapies and/or based on optional evidence of at least one Class I study. Evidence can show some statically modest effect or clinically limited response (partial), or there may be significant issues regarding cost-benefit. There may be substantial disagreement (or potential) among specialists or those responsible for payment and specialists.

Minimum criteria included: studies with norms of age and schooling, applied in elderly and patients with dementia. Any discrepancies were explicitly stated in the event of widely used tests not fulfilling the criteria specified.

The following aspects were considered for the analysis of the instruments for the three modalities of assessment:

- Translation and adaptation.
- Internal consistency.
- Convergent and divergent validity.
- Temporal stability.
- Diagnostic validity (accuracy: sensitivity, specificity and other diagnostic parameters).
- Analysis of sociodemographic influences (age, schooling, gender).

## Functional assessment

Progressive loss in the ability to perform activities of daily living (functional disability) is a primary characteristic for diagnosing dementia. Activities of daily living can be divided into basic (BADL) and instrumental (IADL). BADL are important for self-care and include the ability to carry out personal hygiene, sphincter control and feeding. IADL are more complex and include the ability to prepare meals, carry out domestic chores, manage finances and correspondence, and administer medications.^13^ Functional assessment is useful not only for diagnosing dementia of the Alzheimer type but also for proper guidance of patient and their caregivers and for assessing the effects of pharmacological and non-pharmacological interventions.

At the early stage of dementia due to Alzheimer’s disease, a decline in IADLs is seen and functional assessment with the aim of diagnosis should focus on these aspects by using an interview with an informant or by direct assessment of the patient. Assessing BADL is relevant at more advanced stage of the disease.

Searches employing the terms “activities of daily living” and “Brazil” retrieved 406 articles from the PUBMED database, 72 from LILACS and three on SCIELO. The search terms “activities of daily living” and “Alzheimer’s disease” were also used on the LILACS and SCIELO databases, identifying 23 and three articles, respectively. Studies not involving elderly were excluded as were those which investigated specific clinical conditions (e.g. patients with cardiac disease, chronic pulmonary disease, or spinal cord injuries), focused on aspects of physical mobility or profiles of physical activity, as well as studies employing semi-structured interviews as opposed to scales or standardized questionnaires. The selection yielded 43 articles, among which the instruments used for assessing activities of daily living in elderly and dementia patients in Brazil were identified. In the present analysis, the most frequently used instruments in the studies were the Lawton-Brody^14^ for assessing instrumental activities of daily living and the Katz^15^ scale for assessing basic activities of daily living, Other instruments employed were the Barthel index,^16^ the Functional Activities Questionnaire by Pfeffer et al.,^17^ Functional Independence Measure (FIM),^18^ Informant Questionnaire on Cognitive Decline in the Elderly (IQCODE),^19^ the Disability Assessment for Dementia (DAD),^20^ Bristol Activities of Daily Living Scale (BADLS),^21^ Bayer Activities of Daily Living Scale (B-ADL),^22^ Activities of Daily Living Questionnaire (ADL-Q)^23^ and the Direct Assessment of Functional Status-Revised (DAFS-R).^24^

The Katz scale and Barthel Index were used to assess BADL. The Katz scales was cross-culturally adapted for use in the Brazilian population^25^ (Class II) and is frequently used in studies involving patients with dementia. Similarly, the Barthel index has been validated for the Brazilian population^26^ (Class II), and whose scores were found to correlate with cognitive deficit assessed by the MMSE in the elderly population.^27^ However, no studies were found assessing its application in AD patients. Among the instruments assessing IADL, the IQCODE, the Pfeffer scale and Lawton-Brody scale were widely used in studies on dementia patients in Brazil,^28-31^ but only the IQCODE had been previously validated^28,32^ (Class II). The Lawton scale had undergone a reliability study in a sample of 16 elderly without dementia (Class IV). The Pfeffer scale, despite being extensively used and cited in numerous studies, has not yet been validated.

Other instruments assessed BADL and IADL. The DAD^20,34,35^ (Class III and II studies, respectively), Bayer^22,36,37^ (Class II) and DAFS-R^24,38^ (Class II) scales have corresponding validation studies and rates of diagnostic accuracy for dementia due to AD. The ADL-Q scale had been translated, adapted and analysed for psychometric characteristics^23,39^ (Class II), but had no associated studies in Brazil assessing its diagnostic accuracy for AD. The MIF was validated in Brazil for patients with spinal cord injury^18,40^ (Class II) and was used in studies on elderly groups.^41^ However, only one study analyzing its application in AD patients was identified.^42^ The BADL was used in studies of dementia patients^43,44^ but no studies on its adaptation or validation for use in the Brazilian population were available.

**Recommendations** - For the diagnosis of AD, the use of the IQCODE, DAFS-R, DAD, ADL-Q and Bayer scales can be recommended to assess IADL given they have Class II and III studies validating their use (norm). The Katz scale may be used for assessing BADL in AD patients (norm).
**Note** - It is current practice in our setting to use scales for which there are no validation studies held on the data bases searched, such as the Pfeffer scale^17^ and the BADLS,^21^ pointing to the need for future studies.

## Behavioral assessment

Behavioral and psychological symptoms of Alzheimer’s disease are common during the course of disease evolution, being one of the main reasons for institutionalization, use of medications, high costs of care and family burden.

Several instruments have been developed to systematically assess the neuropsychiatric symptoms of AD. The majority of these scales consist of symptoms rated by informants, typically patient family members and/or caregivers.

Descriptors used:

- Neuropsychiatric Symptoms and dementia or AD.
- Behavioral symptoms and dementia or AD.
- Neuropsychology and dementia or AD.
- Behavioral problems and dementia or AD.
- Behavioral and psychological symptoms (BPSD) and dementia or AD.

According to the review carried out for the search terms above, the most used scales internationally were: Neuropsychiatric Inventory – NPI,^45^ Behavior Rating Scale for Dementia of the Consortium to Establish a Registry for Alzheimer’s Disease (CERAD-BRSD),^46^ Behavioral Pathology in Alzheimer’s Disease Scale (BEHAVE-AD),^47^ and section A of the CAMDEX-R (Cambridge Examination for Mental Disorders of the Elderly – Revised Version).^48^ The Cornell Scale for Depression in Dementia^49^ and the Dementia Mood Assessment Scale (DMAS)^50^ were found for the assessment of depressive symptoms. Cohen-Mansfield Agitation Inventory (CMAI)^51^ is widely used for assessing the broad spectrum of agitation symptoms.

On a national level, 22 articles were found using the same key words, with only 3 articles addressing instrument adaptation and/or validation. Adaptation and/or validation studies were found for the NPI scale,^52^ section A of the CAMDEX-R scale^53^ (Class II), and Cornell^54^ (Class III) scale that fulfilled the minimum criteria for validation.

**Recommendations** - The NPI and CAMDEX scales (Class II or III studies) can be used for the assessment of neuropsychiatric symptoms in AD patients (norm). The Cornell scale can be employed for evaluating depressing symptoms (Class IV)(practice option).

## Brief cognitive screening instruments

Brief screening Instruments not requiring extensive training and that can be applied by a range of health professionals tend to be used in primary care services The descriptors used to search for evidence were: mental status and dementia or AD and Brazil, screening test and dementia or AD and Brazil.

The descriptors applied in the search were mental state AND screening AND dementia AND Brazil. Of all the articles retrieved from the databases on brief instruments (n = 87) applied in Brazil, 29 involved adaptation and/or validation stages of some kind.

The Mini-Mental State Exam (MMSE) was the most used instrument for this purpose and has normative data, test-retest reliability, and diagnostic accuracy as described below.

The MMSE was designed to be an assessment of change in cognitive status of geriatric patients for application in routine clinical practice.^7^ It examines time and spatial orientation, short-term memory (immediate or attention) and recall, calculus, praxia, language and visuospatial skills. The MMSE can be used as a screening test for cognitive loss or as a bedside cognitive instrument.

Another brief test is the Cognitive Abilities Screening Instrument-Short Form: CASI-S)^67^ a scale with a validations study in Brasil.^68^ Some batteries incorporate a section on cognitive assessment without taking much longer to apply, such as the Brief Cognitive Screening Battery (BCSB) and Addenbrooke’s Cognitive Examination-Revised (ACE-R)^69^ which also has associated validation studies for the Brazilian population.^70-73^

**Recommendations** - The Mini-Mental State Exam can be used for the assessment of mental status/cognitive screening in the detection of AD (standard). Other instruments such as the CASI-S, the Brief Cognitive Screening Battery and the Addenbrooke’s Cognitive Examination-Revised can also be used, broadening the scope of cognitive assessment (norm).

## Multi-functional batteries

Multifunctional batteries provide more in-depth assessment but need longer to apply and a specific setting for application.

The search terms neuropsychological tests/neuropsychological battery AND dementia AND validity/applicability/adaptation/sensitivity AND Brazil were employed to assess the situation of multifunctional batteries. Of all the articles retrieved from the databases (n = 83) only 12 assessed aspects of adaptation, reliability or accuracy of specific batteries. The Cambridge Cognitive Examination-Revised: CAMCOG-R),^48^ Alzheimer’s Disease Assessment Scale-cognitive sub-scale: ADAS-COG),^74^ Consortium to Establish a Registry for Alzheimer’s Disease: CERAD),^9^ Mattis Dementia Rating Scale: MDRS)^75^ has corresponding adaptation, reliability or validation studies in Brazil^59,76,77^ (Class II and III).

**Recommendations** - The CAMCOG-R, ADAS-COG, CERAD and MDRS scales are valid for use in the assessment of multifunctional neuropsychology in Alzheimer’s disease (pattern).

## Specific cognitive areas

The selection of the evidence was performed using the PUBMED database with the terms “memory”, “dementia”, “Brazil”, with the limits “Humans”, “English”, Spanish”,”65+ years”,”80+ years” retrieved 58 articles, of which 15 were related to cognitive instruments. Using the same constraints, another search was carried out with the key words “memory”, “elderly”, “Brazil” that yielded 131 articles. This search identified 15 articles that had been previously matched, and seven newly found articles on cognitive instruments. The same procedure was repeated, replacing the word “memory” with “attention” and returned 12 articles, all of which either matched those found on previous searches or were not relevant to the theme of cognitive instruments. The search procedure was repeated using the words “executive function” and subsequently with “visuospatial”, finding 12 and 5 articles, respectively. Three new articles on cognitive instruments were identified – two of which were on executive functions and one on visuospatial functions. In total, 25 Brazilian articles were identified addressing the assessment of cognitive instruments used in the Brazilian elderly population.

On the LILACS database, the search words “memory” and “dementia” were used, identifying a total of 315 matches, 10 of which had previously been located on PUBMED. The search was repeated with “attention test” and “dementia” yielding 24 articles, four of which were new relevant articles.

Using the words “executive function” and “dementia”, seven articles were identified. However, all had previously been retrieved in earlier searches or were not pertinent to the theme. Using the search terms “visuospatial” and “dementia”, two articles were located, which again were not relevant or had been located in earlier searches. The searches were repeated by cross referencing each cognitive function with the word “elder”, returning nine hitherto unidentified articles.

The same search strategies used on LILACS were repeated for the SCIELO database. A search using “memory” and “dementia” produced 57 articles, whereas “memory” and “elderly” found 33 papers, four of which were new. The searches with the words “attention test”, executive function”, “visuospatial” cross-referenced with “dementia” or “elderly” produced no matches. Table 4 summarizes the results of the searches carried out on the databases.

**Table 4:** Summary of results of databases searches for memory function assessment.

Database	Terms used	No. of articles	
		**Retrieved**	**Selected**
PUBMED	Memory × Dementia × Brazil	58	15
	Memory × Elderly × Brazil	131	7
	Attention × Dementia × Brazil	12	0
	Executive function × Dementia × Brazil	12	2
	Visuospatial × Dementia × Brazil	5	1
LILACS	Memory × Dementia	315	10
	Attention test × Dementia	24	4
	Executive function × Dementia	8	0
	Visuospatial × Dementia	2	0
SCIELO	Memory × Dementia	57	2
	Memory × Elderly	33	2
	Attention test × Dementia (or elderly)	0	0
	Executive function × Dementia (or elderly)	0	0
	Visuospatial × Dementia (or elderly)	0	0

The analysis of articles selected and discussion among panel members led to a consensus on the scientific evidence supporting the use of the previously studied cognitive instruments in Brazil. The resultant recommendations for clinical use are outlined below under each specific cognitive area.

## Memory

Memory problems are the most important component in cognitive investigation for inclusion in the diagnostic criteria of AD. Patients with AD manifest early deficits in the acquisition of new information and loss of information during later recall. The tests recommended for assessing memory include immediate and delayed recall of words, and concrete or abstract figures in verbal and visual modalities.

For assessing verbal memory, the Rey Auditory Verbal Learning Test^78-83^ (RAVLT) and the word list from the cognitive battery of the Consortium to Establish a Registry for Alzheimer’s Disease (CERAD)^59,84,85^ are tests which meet the minimum validation requirements having significant normative data and wide application in the Brazilian population.

For assessing visual memory, the 10 figure test from the Brief Cognitive Screening Battery (BCSB)^71,86-90^ and the geometric figure recall task from the CERAD battery,^59,84,85^ are tests which meet the minimum validation requirements, having significant normative data and wide application in the Brazilian population.

Rey’s Complex Figure Test assesses both visuoconstruction and non-verbal memory. The validation for the Brazilian elderly population was recently constructed, broadening the potential scope of applications underway in our milieu.^91,92^

The Rivermead Behavioural Memory Test (RBMT),^93,94^ the Short Cognitive Performance Test (SKT)^95,96^ and the Logical Memory subtest of the Wechsler Memory Scale - III (WMS-III)^55,97^ were preliminarily validated for the Brazilian population through the growing number of applications of the test in the country.

**Recommendations** - The RAVLT tests, ten figures from the BCSB, and the word list and figure recall of the CERAD battery should be used for assessing memory in the diagnosis of AD – Class II and III studies (guideline). Rey’s Complex Figure should be used while considering its limitations in clinical value (practice option).
**Notes** - Although widely applied in the Brazilian population, the Figure Object Memory Evaluation (FOME),^94,97^ the Selective Reminding Test and the Visual Reproduction test of the WMS-III battery still lack validation studies for Brazil.

## Attention

Attention can be compromised in early phases of AD. Patients with AD experience deficits in all types of attention, with greatest difficulty in switching attentional focus.

In the auditory modality, the recommended tests include the forward (attentional abilities) and backward (executive and attentional control abilities) digit span test from the Wechsler Adult Intelligence Scale III (WAIS-III) test battery. The WAIS-III has been translated and adapted to Portuguese and the norms for the Brazilian population are available and extensively used.^98,99^

In the visual modality, the Trail Making test included two modalities – part A recruits attention and part B divided attention. Many Brazilian studies involve the use of the Trail Making test. Although not validated for use in Brazil, some studies report comparisons among clinical groups and norms for age groups organized by level of schooling.^91,94,100,101^

**Recommendations** - The forward/backward Digit Span subtest should be used to assess attention in AD diagnosis (norm).

## Executive functions

Deficit in executive functions – election of objectives, planning, organizing responses and monitoring encompass the group of alterations seen in AD. The Clock Drawing Test (CDT) is able to assess multiple cognitive domains including semantic memory, visuoconstruction and executive functions, given that good performance requires planning and monitoring of actions. The CDT is validated for use in Brazil and has an established cut-off score for AD applicable to the Brazilian population.^102-108^

The Wisconsin Card Sorting Test is considered the classic instrument for assessing executive functions, since it measures understanding of rules for card combinations and the ability to change the rules during the course of the task. The test has defined norms for the elderly Brazilian population (application manual), although no validation studies were available in the databases consulted. Studies using the test in the Brazilian elderly were found^91,109^ while another study suggested the method of application, albeit manual or computer-based, had no influence on outcomes among elderly Brazilians.^110^

The executive aspect is prominent in the verbal fluency tests. In Brazil, normative data are available for phonemic fluency – age group, age and schooling^111,112^ and for semantic fluency – age group and schooling^113-116^ and from applications of these instruments in clinical studies.

Also with regard to executive function, the Similarities subtest of the WAIS-III battery for assessing abstract thought was validated with norms established for the Brazilian aged population. The Stroop test, although frequently used in our milieu, has not yet been formally validated for the Brazilian population.^91,109^

**Recommendations** - The CDT and verbal fluency tests (phonemic and semantic) can be used for assessing executive functions in AD (guidelines). The Similarities subtest of the WAIS-III battery can be used for assessing abstraction ability (practice option).
**Notes** - The Executive Interview EXIT-25,^117,118^ Battery for Behavioral Assessment of the dysexecutive Syndrome (BADS)^119,120^ and the Frontal Assessment Battery (FAB)^121^ are currently undergoing the initial stage of validation in Brazil.

## Visuoperceptual and constructive abilities

These abilities are impaired in the late stages of AD and no tests have been fully validated in Brazil for assessing these cognitive aspects. For assessing visuoconstructive abilities, the Figure Copying subtest of the CERAD battery has preliminary validation.^59,84,85^ The CDT cited above is also used to assess visuoconstructive ability.^102-106,108^ The Cubes subtest of the WAIS-III battery can also be used since it has been validated for Brazil.^97-99^ Rey’s Complex Figure which, although the focus of few studies in the Brazilian elderly population,^91,92^ may be utilized to assess planning ability during the execution of a visuoconstructive task.

Another option for assessing visuoperceptual ability is the Matrix Reasoning subtest of the WAIS-III battery, validated for use in Brazil,^97-99^ a test which is equivalent to the Raven’s Colored Progressive Matrices test. This latter test has been validated for use in children and may also be employed in the assessment of elderly. However, no studies on this instrument were found on the databases searched.

**Recommendations** – The Figure copying and CDT tests should be used for assessing constructive abilities in AD (norm).

### SUGGESTED MINIMUM PROTOCOL

A proposed minimum protocol for assessing specific cognitive functions in dementia of the Alzheimer type is given in Table 5.

**Table 5:** Minimum protocol proposed by Consensus for assessing specific cognitive areas for diagnosis of dementia of the Alzheimer type.

Cognitive domains	Brief assessment (30 minutes or less)	Expanded assessment (approx. 1 hour)
Memory	10 figures from BCSB 10 words from CERAD	RAVLT Logical Memory (WMS-III) CERAD Figure Recall Rey's Complex Figure
Attention and Executive functions	Forward and Backward Digit Span Verbal Fluency Animals CDT	Similarities (WAIS-III) FAS Wisconsin Card Sorting Test Trail-making A and B
Language	Boston Naming	Boston Battery Arizona Battery
Visuoperceptual and visuoconstruction	CDT	Reasoning Matrix (WAIS-III) Figure Copying from CERAD Rey's Complex Figure

## Language

Language difficulties are acknowledged as an early sign of AD, particularly naming difficulties.

In relation to language, the database searches used the terms language × Alzheimer’s disease, and found no published articles on the topic. The closest terms were language and cognition which identified 3 (three) publications none of which were relevant for the theme in question. Similarly, a search for “language × Alzheimer’s disease” on LILACS found no published articles. The closest uniterms on the theme of interest were language and cognition. A total of 92 articles were found on PUBMED, two of which were Brazilian publications pertinent to the theme.

On PUBMED, studies were identified on *Boston Naming*,^122^ undoubtedly the most commonly used test for this purpose. In Brazil, there is an adaptation and norms study for different age and schooling groups involving a large sample, with suggested expected performance values for different age groups, genders and educational levels.^123^ The verbal fluency tests are also used with the aim of detecting lexical compromise in early AD. Studies in Brazilian Portuguese sought to determine the effects of age, schooling and gender on semantic verbal fluency.^113,116^

The Arizona battery is recommended for assessing the language/memory interface in dementia and has been the subject of preliminary validation and accuracy studies The full protocol of the Boston battery has a similar validation status.^124,125^ Other comprehensive batteries for assessing language were applied in samples of healthy individuals, enabling the determination of performance cut-off scores. This is also the case for the Beta-MT Battery.^126^ All of the cited studies are Class III level of evidence.

**Recommendations** - The assessment of language for diagnosing AD should be carried out using the Boston Naming or semantic verbal fluency test (guidelines). Patients showing impairment on the recommended tests should undergo more rigorous assessment by the Arizona, Boston or Beta MT batteries (practice option).

## Clinical dementia rating scale

The clinical dementia rating scale – CDR^127^ has associated Class I and II validation studies in Brazil.^128-130^

**Recommendations** - In order to assess dementia in Alzheimer’s disease, as well as classify patients according to disease stage, the clinical dementia rating (CDR) can be used (standard).

## Final comments

In view of the profile of the instruments in use for cognitive, functional and behavioral evaluations and also the validation studies which cover the majority of the psychometric characteristics of many tests and scales, it can be recommended that further studies of this nature be fostered in Brazil and run with the support of research funding bodies.

It should be stressed that the analyses and recommendations outlined apply specifically to the diagnosis of Alzheimer’s disease. Mild cognitive impairment (MCI), in its broader meaning or in reference to the more specific precursor to Alzheimer’s disease (according to diagnostic guidelines) warrants a separate review to determine the value of the tests and scales available for cognitive, functional and behavioral assessment. The challenge of reaching a consensus for the diagnosis of MCI remains.

## Figures and Tables

**Table 3:** Sensitivity and Specificity of Mini Mental State Exam for detecting dementia.

Study	Sample	Cut-off	Sensitivity	Specificity
Chaves and Izquierdo,1992^55^	31 patients with dementia, 31 patients with major depression and 22 healthy controls	24	96%	68%
Bertolucci et al., 1994^56^	94 patients with cognitive impairment and 530 adult controls	Illiterates: 13 Schooling <8 years: 18 Schooling ≥8 years: 26	82.4% 75.6% 80%	97.5% 96.6% 95.6%
Almeida, 1998^57^	211 inds aged ≥60 years Dementia by CID-10	Illiterates: 19 Schooled: 23	80% 84%	71% 60%
Caramelli et al., 1999^58^	Population-based sample, 1656 elderly >64 ys, 570 illiterates, 188 with dementia - 10^th^ , 25^th^ and 50^th^ percentiles defined	Illiterates: 15, 18, 20 Schooling: 1-3 years: 21, 23, 25 4-7 years: 22, 25, 26 >7 years: 25, 26, 28	-	-
Bertolucci et al., 2001^59^	85 healthy elderly and 43 patients with AD	26	97.6%	75.3%
Brucki et al., 2003^60^	433 normal individuals	Illiterates: 20 By schooling 1-4 years: 25 5-8 years: 26.5 9-11: 28 >11: 29	-	-
Laks et al., 2003^61^	341 elderly	Younger old: 19.9 Older old: 16.9 Illiterates: 17.1 Schooled: 22.3	-	-
Lourenço and Veras, 2006^62^	303 elderly general outpatients 78 with dementia by DSM-IV	Illiterates: 18/19 Schooled: 24/25	73.5% 75%	73.9% 69.7%
Laks et al., 2007^63^	870 elderly from community	Younger old Illiterates: 19.5 1-8 years: 23.9 Older old Least schooled: 18.1 Most schooled: 23.8	-	-
Castro-Costa et al., 2008^64^	1558 individuals (≥60 ys) from community	General 5^th^ Percentile: 14 95^th^ Percentile: 22 60-64 years: 5^th^ Percentile: 17 95^th^ Percentile: 24 ≥65 years: 5^th^ Percentile: 13 95^th^ Percentile: 16	-	-
Lourenço et al., 2008^65^	306 individuals ≥65 ys, outpatients105 sub-sample of 1 week retest	PC: 23/24	-	-
Kochhann et al., 2010^66^	162 patients with dementia806 healthy elderly	Illiterates: 21 Low schooling: 22 Medium: 23 High: 24	93% 87% 86% 81%	82% 82% 87% 87%
